# Phase II/III trial of hyperimmune anti-COVID-19 intravenous immunoglobulin (C-IVIG) therapy in severe COVID-19 patients: study protocol for a randomized controlled trial

**DOI:** 10.1186/s13063-022-06860-2

**Published:** 2022-11-08

**Authors:** Shaukat Ali, Elisha Shalim, Farah Farhan, Fatima Anjum, Ayesha Ali, Syed Muneeb Uddin, Faisal Shahab, Mustafa Haider, Iqra Ahmed, Mir Rashid Ali, Sadaf Khan, Sadia Rao, Kabeer Guriro, Saud Elahi, Muhammad Ali, Tehreem Mushtaq, Muneeba Ahsan Sayeed, Sheikh Muhammad Muhaymin, Shoba Luxmi, Saifullah, Saeed Qureshi

**Affiliations:** 1 Dow College of Biotechnology, Dow University of Health Sciences, Karachi, Pakistan; 2 Department of Biotechnology, University of Karachi, Karachi, Pakistan; 3 Dow Research Institute Of Bio-Technology And Bio-Sciences (DRIBBS), Dow University of Health Sciences, Karachi, Pakistan; 4 Sindh Infectious Disease Hospital and Research Center, Karachi, Pakistan; 5 Dow University Hospital, Dow University of Health Sciences, Karachi, Pakistan

**Keywords:** SARS CoV-2, Pooled convalescent plasma, Polyclonal antibodies, Anti-COVID-19 IVIG, Passive immunization, Randomized controlled trial, Hyperimmune, Intravenous, Pneumonia, Novel coronavirus

## Abstract

**Background:**

COVID-19 poses a global health challenge with more than 325 million cumulative cases and above 5 million cumulative deaths reported till January 17, 2022, by the World Health Organization. Several potential treatments to treat COVID-19 are under clinical trials including antivirals, steroids, immunomodulators, non-specific IVIG, monoclonal antibodies, and passive immunization through convalescent plasma.

The need to produce anti-COVID-19 IVIG therapy must be continued, alongside the current treatment modalities, considering the virus is still mutating into variants of concern. In this context, as the present study will exploit pooled diversified convalescent plasma collected from recovered COVID-19 patients, the proposed hyperimmune Anti-COVID-19 intravenous immunoglobulin (C-IVIG) therapy would be able to counter new infectious COVID-19 variants by neutralizing the virus particles. After the successful outcome of the phase I/II clinical trial of C-IVIG, the current study aims to further evaluate the safety and efficacy of single low dose C-IVIG in severe COVID-19 patients for its phase II/III clinical trial.

**Methods:**

This is a phase II/III, adaptive, multi-center, single-blinded, randomized controlled superiority trial of SARS-CoV-2 specific polyclonal IVIG (C-IVIG). Patients fulfilling the eligibility criteria will be block-randomized using a sealed envelope system to receive either 0.15 g/Kg C-IVIG with standard of care (SOC) or standard of care alone in 2:1 ratio. The patients will be followed-up for 28 days to assess the primary and secondary outcomes.

**Discussion:**

This is a phase II/III clinical trial evaluating safety and efficacy of hyperimmune anti-COVID-19 intravenous immunoglobulin (C-IVIG) in severe COVID-19 patients. This study will provide clinical evidence to use C-IVIG as one of the first-line therapeutic options for severe COVID-19 patients.

**Trial registration:**

Registered at clinicaltrial.gov with NCT number NCT04891172 on May 18, 2021.

## Administrative information

Note: the numbers in curly brackets in this protocol refer to SPIRIT checklist item numbers. The order of the items has been modified to group similar items (see http://www.equator-network.org/reporting-guidelines/spirit-2013-statement-defining-standard-protocol-items-for-clinical-trials/).

**Table:** 

Title {1}	Phase II/III trial of Hyperimmune Anti-COVID-19 Intravenous Immunoglobulin (C-IVIG) therapy in Severe COVID-19 Patients: Study protocol for a Randomized Controlled Trial
Trial registration {2a and 2b}.	ClinicalTrials.gov identifier (NCT number): NCT04891172
Protocol version {3}	Protocol version 3, October 12, 2022
Funding {4}	Financial, material, human resource and other support is provided by Dow University of Health Sciences (DUHS), Karachi, Pakistan.
Author details {5a}	SPIRIT guidance: Affiliations of protocol contributors. 1. Dow College of Biotechnology, Dow University of Health Sciences, Karachi, Pakistan 2. Dow University Hospital, Dow University of Health Sciences, Karachi, Pakistan 3. Sindh Infectious Disease Hospital and Research Center, Karachi, Pakistan 4. Department of Biotechnology, University of Karachi, Karachi, Pakistan
Name and contact information for the trial sponsor {5b}	Dow University of Health Sciences, Ojha Campus, Gulzar-e-Hijri Scheme-33, Suparco Road, Karachi, Pakistan Phone: +92 21 38771111
Role of sponsor {5c}	Financial, material, human resource and other support is provided by Dow University of Health Sciences (DUHS), Karachi, Pakistan. The sponsor has no role in study design, collection, analysis, and interpretation of data or in writing the manuscript.

## Introduction

### Background and rationale {6a}

On March 11, 2019, the World Health Organization declared COVID-19 as a pandemic [1]. Severe acute respiratory syndrome coronavirus 2 (SARS-CoV-2) is the causative agent of this systemic disease. COVID-19 has been categorized as a biphasic illness with an early phase of viral replication and second phase directed by host immune response [2]. This second phase may lead to severe and critical COVID-19 cases progressing towards a life-threatening multiple organ dysfunction, characterized by refractory hypoxemia due to acute respiratory distress syndrome (ARDS) [3].

Despite the global and continuous efforts of researchers to combat COVID-19 including provision of vaccines to the mass population, there is still a surge in COVID-19 cases due to emergence of new variants of the SARS CoV-2 making vaccines less effective. This emphasizes the need to develop broadly protective interventions against the evolving pandemic [4]. Convalescent plasma (CP) from recovered patients has been used previously as a passive immunotherapy in epidemics associated with coronaviruses, SARS CoV-1 in 2003, Middle East respiratory syndrome (MERS) in 2012, and also in the current COVID-19 pandemic [5]. Intravenous immunoglobulin (IVIG) extracted from CP is recommended for the treatment against a variety of inflammatory, infectious, autoimmune, and viral diseases including SARS and MERS [6, 7]. IVIG helps in regression of disease from multiple fronts including virus neutralization and immunomodulation through anti-cytokine activity and prevention against other superimposed bacterial infections due to the polyclonal formulation of the drug [8–10]. Hyperimmune anti-COVID-19 intravenous immunoglobulin (C-IVIG) is an unexplored therapy amidst the rapidly evolving spectrum of medical therapies for COVID-19 and is expected to counter the three most life-threatening consequences of COVID-19 including lung injury by the virus, cytokine storm, and sepsis [11].

Despite great interest, due to lack of availability of clinical evidence for the safety and efficacy of hyperimmune IVIG in COVID-19 patients, the use of hyperimmune intravenous immunoglobulin as one of the first-line therapeutic options against COVID-19 has been limited. In this context, an adaptive phase II/III, multicenter center, single-blinded superiority trial has been designed to assess the safety and efficacy of single dose of hyperimmune anti-COVID-19 intravenous immunoglobulin (C-IVIG) in severe COVID-19 patients after observing safety and efficacy of C-IVIG in phase I/II clinical trial of severe and critical COVID-19 patients [11].

### Objectives {7}

This trial aims to investigate the safety and clinical efficacy of hyperimmune anti-COVID-19 Intravenous Immunoglobulin (C-IVIG: 5% liquid formulation), on severe COVID-19 patients.

### Trial design {8}

**Fig. 1:**
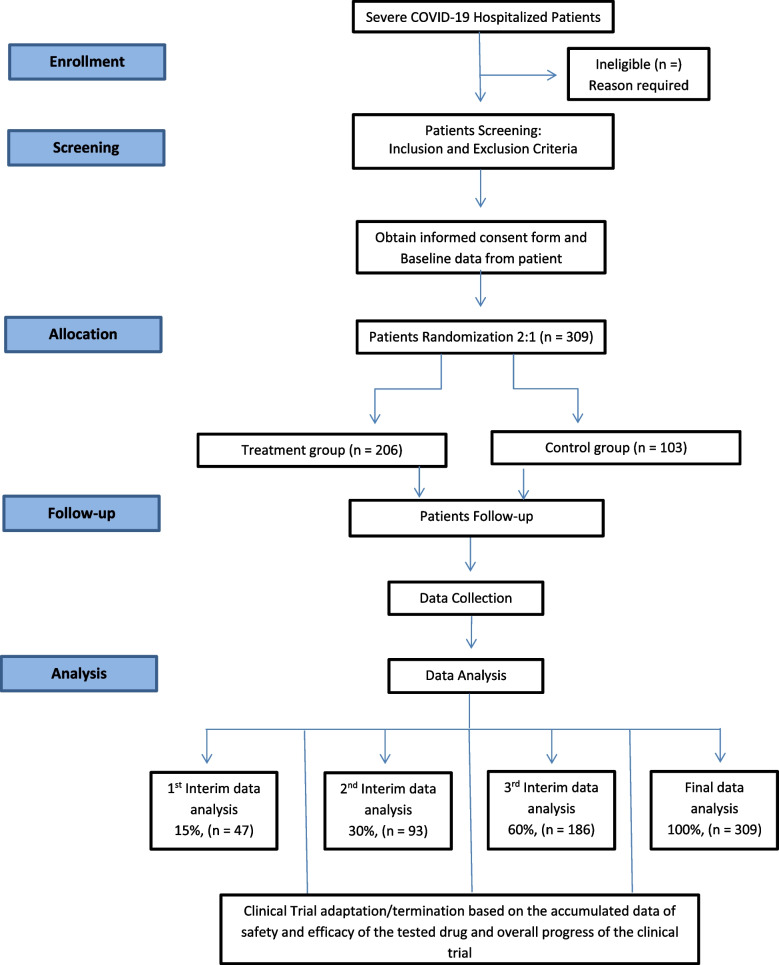
Consort flow diagram of the trial design

This is an adaptive, phase II/III, multi-center, randomized controlled, single-blinded superiority trial with parallel group design to assess the safety and efficacy of single dose of C-IVIG in patients with symptoms of severe COVID-19. Eligible participants will be randomly assigned in 2:1 ratio (206 tests: 103 controls) by block randomization method to receive C-IVIG plus standard of care (intervention group) or only standard of care (control group). The adaptive study design refers to adapting the strategy of trial based on interim analysis at three particular time points [12]. Outcome of the interim analysis will lead to making decision for terminating trial in case of serious adverse events or selecting a higher C-IVIG dose regime in case selected dose of C-IVIG (0.15g/Kg) not found efficacious. A flow chart of the trial design is shown in Fig. 1.

## Methods: participants, interventions, and outcomes

### Study setting {9}

The trial will be conducted in Karachi, Pakistan at Dow University of Health Sciences (Ojha Campus) and Sindh Infectious Disease Hospital and Research Center (SIDH).

### Eligibility criteria {10}

#### Participant selection

A total of 309 participants from both male and female gender will be recruited according to the following inclusion and exclusion criteria.

Inclusion criteria for participant:

- Study participants should be above 18 years of age
- Have positive SARS-CoV-2 PCR on nasopharyngeal and/or oropharyngeal swabs
- Classified as severe COVID-19 according to WHO guideline (5L-15L of oxygen requirement on face mask (FM), Non-rebreather mask (NRM)
- Have signed the consent to participate in the study

Exclusion criteria for participants:

- Participants will be excluded if they have critical COVID-19 [non-invasive ventilation (HFNC, BiPAP, CPAP) and invasive ventilation]
- Pregnant females
- Previous allergic reaction to immunoglobulin treatment
- Known case of any autoimmune disorder
- Chronic renal disease
- Known case of thromboembolic disorder
- Aseptic meningitis

#### Who will take informed consent? {26a}

Consent will be taken by the Clinical Research Associate, or the treating physician on a document designed as per guidelines of Drug Regulatory Authority of Pakistan (DRAP). The participant itself or first-degree relative is eligible to provide consent.

#### Additional consent provisions for collection and use of participant data and biological specimens {26b}

Provisions to collect and use participant’s data and biological specimens are mentioned in informed consent form.

### Interventions

#### Explanation for the choice of comparators {6b}

The intervention group will receive a single dose of C-IVIG (0.15 g/Kg) along with standard hospital care and participants will be assigned in a sequential manner. C-IVIG is a 5% concentrated preparation of anti-SARS-CoV-2 antibodies purified from fractionation of pooled convalescent plasma of COVID-19 survivors [13]. Hyperimmune C-IVIG was selected for evaluation as it has not yet been used as a treatment modality for severe COVID-19 patients, although multiple clinical trials to assess convalescent plasma and immunoglobulins as therapeutic options for COVID-19 are being conducted globally. C-IVIG when infused in COVID-19 patients is expected to regulate disease progression via multiple mechanisms including SARS-CoV-2 neutralization, immunomodulation to prevent cytokine storm, and prevention of superimposed bacterial infection (sepsis) due to presence of polyclonal antibodies against other endemic pathogens [11]. C-IVIG is assumed to contain polyvalent antibodies against SARS-CoV-2 and other co-existing infections with no drug-related adverse events observed, hence a potential and safer treatment modality for COVID-19. The lower dose of C-IVIG (0.15 g/Kg) was selected due to its proven safety and efficacy for severe COVID-19 patients in phase I/II clinical trial [11]. The control group will receive standard hospital care only. Participants will be assigned in a sequential manner in both groups.

#### Intervention description {11a}

Intervention group will receive a single dose of 0.15 g/kg body weight C-IVIG along with standard care. Control arm will receive standard hospital care only. The dose will be administered intravenously with a flow rate of 20 ml/h in the first hour and 25 ml/h till the dosage end. Standard hospital care includes airway support, anti-viral medication, antibiotics, fluid resuscitation, hemodynamic support, steroids, painkillers, and anti-pyretic. Standard of care will ensure blinding because as part of the usual hospital care severe COVID-19 patients is given multiple infusion, and hence, it will not be possible for patients in intervention group and control group to discriminate between standard of care and C-IVIG infusion.

#### Criteria for discontinuing or modifying allocated interventions {11b}

Modification of the allocated intervention will be based on the planned interim analysis. Outcome of the interim analysis will lead to making decision regarding selection of higher C-IVIG dose in case of currently selected 0.15 g/kg dose not found efficacious. The higher dose of C-IVIG will be selected from the dosage investigated in previous phase I/II clinical trial [11]. The C-IVIG infusion will be discontinued if any serious adverse event occurs or discontinued on patient request and the patient will be excluded from the study.

#### Strategies to improve adherence to interventions {11c}

It is not applicable because the drug will be given as a single dose intravenously by respective healthcare professionals.

#### Relevant concomitant care permitted or prohibited during the trial {11d}

Standard hospital care including all concomitant care and interventions are permitted.

#### Provisions for post-trial care {30}

No provisions are made for ancillary and post-trial care.

#### Outcomes {12}

Primary outcome:

- 28-day mortality [time frame: 28 days]
- All-cause mortality of participants will be monitored for 28 days to assess the safety and efficacy of C-IVIG

Secondary outcomes:

- Immediate (within 24 h) or serious adverse events (throughout the hospital stay) [time frame: 28 days]
- Clinical status according to 7 category ordinal Scale [time frame: 28 days (till hospital stay)
  1. Not hospitalized and no limitations of activities
  2. Not hospitalized, with limitation of activities, home oxygen requirement, or both
  3. Hospitalized, not requiring supplemental oxygen
  4. Hospitalized, requiring any supplemental oxygen
  5. Hospitalized, requiring noninvasive ventilation or use of high-flow oxygen devices
  6. Hospitalized, receiving invasive mechanical ventilation
  7. Death
- Change in Horowitz index (PaO_2_/FiO_2_ ratio use to assess lung function) during hospital stay [time frame: 28 days]
- Change in C-reactive protein (CRP) levels, radiological findings, IL-6 level, and anti-SARS-CoV-2 antibody levels [time frame: 5 days]

#### Participant timeline {13}

The timeline of enrollment and intervention is given in Table 1.

**Table 1:**
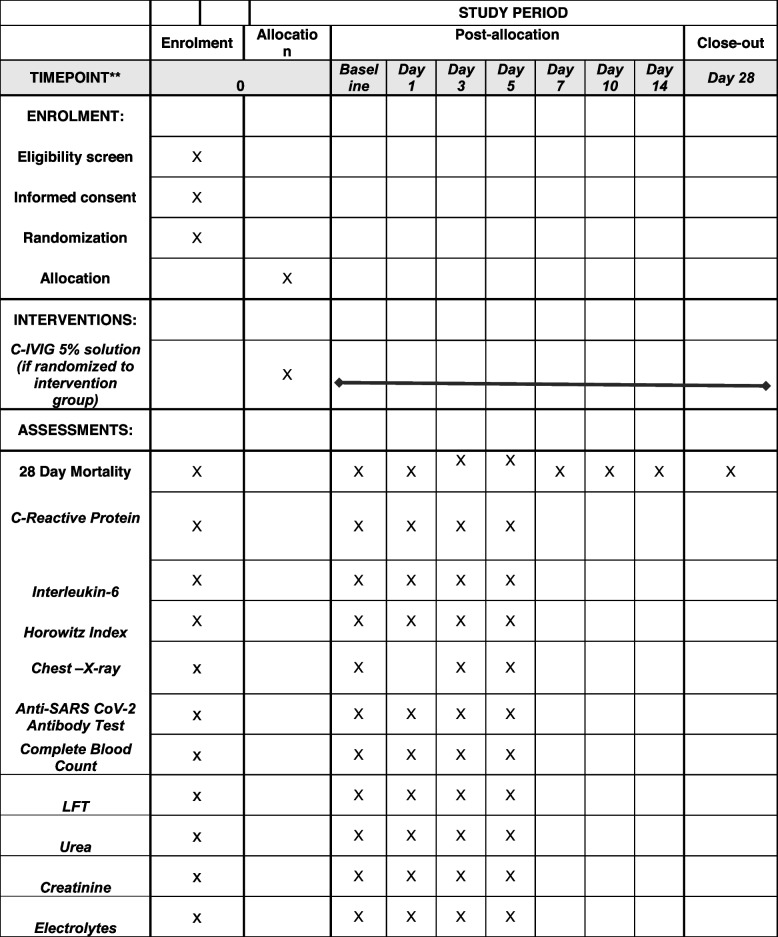
Time schedule of enrolment, interventions, assessments, and visits for participants

#### Sample size {14}

Three hundred nine participants will be enrolled in the study. Sample size was determined by taking proportion of survival in intervention group (P1) = 75%, proportion of survival in control group (P2) = 40%, power of test (1-β) = 90%, with the sample ratio 2:1 (test to control), superiority margin 0.15, and drop rate 30%. The calculated total sample size is 309 patients, 206 tests and 103 control. The calculated sample size was determined with the help of online sample size calculator (www.riskcalc.org) taking 99% confidence level (11).

#### Recruitment {15}

Patients will be recruited from Dow University Hospital, and Sindh Infectious Disease Hospital who will be hospitalized with severe COVID-19 pneumonia.

### Assignment of interventions: allocation

#### Sequence generation {16a}

Randomization will be stratified by site and will be in permuted blocks and the randomized sequence allocation will only be accessible to the site investigator. The randomization schedule will be generated by an independent statistician.

#### Concealment mechanism {16b}

Eligible participants are randomly assigned in 2:1 ratio (206 tests to 103 controls) by sequentially numbered opaque sealed envelopes using block randomization method, either to receive C-IVIG plus SOC (intervention group) or only SOC (control group). A randomization list is generated by a statistician unrelated to this study, while the study personnel will be unaware of the sequence of assignments. At the time of randomization, the study personnel will receive a sealed opaque envelope with assignment to intervention or control group.

#### Implementation {16c}

Participants will be enrolled by the clinical research associate or by treating physicians of each study site. Participants will be assigned to interventions by investigators according to a randomization list.

### Assignment of interventions: blinding

#### Who will be blinded {17a}

This is a single-blinded trial in which trial participants are blinded.

#### Procedure for unblinding if needed {17b}

Unblinding is not needed because the trial is single blinded and only patients will be blinded. Unblinding will only be permissible by study investigators when patient selects to leave against the medical advice (LAMA) to seek treatment in another hospital and the information is important for the safe management of the patient.

### Data collection and management

#### Plans for assessment and collection of outcomes {18a}

Outcome, baseline, and other trial data will be collected by the study investigators. The clinical research associate will check the data. Nasopharyngeal/oropharyngeal swabs and blood samples will be sent to DUHS laboratory, where tests will be performed according to laboratory standard operating procedures (SOPs). Data reports can be found on HMIS (Hospital Management information system) and will be collected on clinical research forms (CRF).

#### Plans to promote participant retention and complete follow-up {18b}

Designated personnel from the trial staff will be appointed to ensure the participant retention and complete follow-up during the participant’s hospital stay and after discharge as well. After a participant’s hospital discharge, participants will be followed on call. The participant can withdraw from the trial at any point. The decision will be communicated with the study investigator; the clinical research associate should be explaining the importance of staying in the trial for the full duration of follow-up. For withdrawal, there is a form in CRF that will be filled by the clinical research associate with reasoning why the participant is withdrawing from the trial. If the participant will be lost to follow-up, the data that will be collected till the time of loss to follow-up will be assessed and used in the analysis.

If the patient will withdraw due to AEs, the clinical associate or the treating physicians will closely monitor the follow-up of AEs until the participants return to the baseline state or the participant condition returns to normal. Then the AEs will be reported to the study investigators.

#### Data management {19}

Data will be collected from the online Hospital Management Information System (HMIS) and hospital record files from all clinical trial sites. All relevant data from hospital files will be manually entered in record sheets and CRFs by designated project personnel. The data from record sheets and HMIS will then be transferred to MS Excel sheets. The data files will be accessible to the appointed statistician for data analysis and interpretation.

#### Confidentiality {27}

The hospital record files and HMIS software will be accessible to designated members of the clinical trial team only. All data files will be secured at the office of Clinical Trial Unit (CTU), Dow University of Health Sciences.

#### Plans for collection, laboratory evaluation and storage of biological specimens for genetic or molecular analysis in this trial/future use {33}

Biological specimens of hospitalized patients will be collected and sent to the laboratory by on-duty staff according to the given schedule and as recommended by the treating physician. Discharged patients will be contacted via phone call and their samples will be collected by the study investigators. All samples will ultimately be sent to the laboratory or at predetermined storage conditions.

### Statistical methods

#### Statistical methods for primary and secondary outcomes {20a}

Patients will be randomized at a ratio of 2:1 to receive either C-IVIG with standard of care or standard of care only. The data to be analyzed will be blinded using the SPSS software 24.0. All randomized study participants will be included in the intention-to-treat population and all participants completing the study period will be analyzed (complete case analysis). Normality of continuous data will be checked by the Shapiro-Wilk test, and parametric or non-parametric tests will be applied accordingly. Continuous variables will be presented as mean (± SD) or median (interquartile range), and categorical variables will be presented as percentages. In the primary analysis strategy, we will use the Kaplan–Meier curve (Breslow test) that compares the time to reach the primary end point in the trial groups. Hypothesis testing of outcomes for the assessment of safety and efficacy parameters will be conducted by Mann-Whitney *U* test or independent *t*-test. Categorical data will be compared with chi-square test, and value of the two-sided Fisher exact test will be recorded. An estimate of the relative risk and 95% confidence interval will also be reported. Hypothesis testing will be conducted at a significance level of 0.05.

#### Interim analyses {21b}

An interim analysis will be performed after recruitment of 15%, 30%, and 60% participants in the trial. It will assess primary outcomes of the study particularly focusing on the safety and efficacy of the infused drug. Safety of the intervention will be monitored in terms of any immediate or serious adverse event that can be observed after the administration of the drug. The efficacy of the intervention will be noted in terms of the patient's improvement in clinical status as compared to the control group.

#### Methods for additional analyses (e.g., subgroup analyses) {20b}

No subgroup analyses have been planned for this trial.

#### Methods in analysis to handle protocol non-adherence and any statistical methods to handle missing data {20c}

To handle the missing data multiple imputation will be used or complete case analysis will be used. The primary analysis will use the intention-to-treat principle.

#### Plans to give access to the full protocol, participant level-data, and statistical code {31c}

The datasets analyzed during the current study and statistical codes are available from the corresponding author on reasonable request, as is the full protocol.

### Oversight and monitoring

#### Composition of the coordinating center and trial steering committee {5d}

The study will be conducted in Dow University Hospital, Karachi, Pakistan, and Sindh infectious disease Hospital, Karachi, Pakistan.

### Composition of the data monitoring committee, its role and reporting structure {21a}

#### Adverse event reporting and harms {22}

Adverse events (AE) and serious adverse events (SE) will be observed and reported from the time of patient’s enrollment in the study till the outcome day, i.e., 28 days. AE/SE will be reported in the clinical research forms which include the time of the AE/SE medications, severity, duration of the AE, and the outcomes after AE. The relevant information of adverse events, after investigating if it is drug related or not, will be reported within 24 h to the principal investigator and to the National Drug Authorities.

#### Frequency and plans for auditing trial conduct {23}

The Clinical Research Organization (CRO) “Institute of Biological and Biochemical and Pharmaceutical Sciences (IBBPS), DUHS, Karachi” will be responsible for the auditing and monitoring of the trial. Audits may be conducted at any time during or after the study completion.

#### Plans for communicating important protocol amendments to relevant parties (e.g., trial participants, ethical committees) {25}

The modification/amendment in the protocol of this trial will be informed to the national authorities and regulators by the principal investigator. The changes will be then implemented after the approval from the authorities. Protocol amendments will also be done on trial registries such as clinicaltrial.gov and will be communicated to the journal where trial protocol has been published. After any protocol amendment, the informed consent form and any other written information provided to the subjects will be updated as necessary.

#### Dissemination plans {31a}

The results will be reported to the national authorities such as National Bioethics Committee (NBC) and Drug Regulatory Authority of Pakistan (DRAP) and will be published in the reputed scientific journals, to communicate trial results to participants, healthcare professionals, the public, and other relevant groups.

## Discussion

This phase II/III clinical trial is an adaptive, randomized controlled, multi-center, single-blinded superiority trial of SARS-CoV-2 specific polyclonal IVIG (C-IVIG) versus standard of care for patients hospitalized with severe COVID-19. Single dose of C-IVIG in combination with the standard of care was found to be both safe and efficacious in our previous clinical trial phase I/II while increasing the survival rate and reducing the risk of disease progression [13]. There were no significant changes observed when different doses of C-IVIG were compared in the phase I/II; however, it was found that C-IVIG dosage of 0.15 g/kg have significantly improved the therapeutic response in severe COVID-19 patients. These results warrant the phase II/III clinical trial study of C-IVIG, in order to evaluate its safety and efficacy in large sample size by comparing outcomes (28-mortality, hospital stay and drug-related adverse events) of C-IVIG along with SOC in intervention group and SOC only in comparator group provided during the hospital stay.

Study participant selection for this study is based on the results of our previous clinical trial where severe patients had 100% survivability and early hospital discharge than critical patients and comparator group. We evaluated that hospital stay, reliance on mechanical ventilation, and burden on hospital could be reduced if C-IVIG is administered to the severe COVID-19 patients by delaying the disease progression.

This is an adaptive clinical trial to investigate the risk-benefit analysis of C-IVIG administration for severe COVID-19 at first and second interim of the study. Major outcome variables to be assessed for interim analysis are adverse events, 28-day mortality, and change in clinical status of the patients. Fate of this clinical trial is based on whether the C-IVIG is safe and efficacious. If the treatment modality is found to be safe but not efficacious as compare to comparator group, we might select different dosage of C-IVIG for further study participants; if interim analysis indicate association of serious adverse events with administration of C-IVIG or with significant high mortality ratio in intervention group than comparator group or if incidence rate of COVID-19 is low till the study completion date, then this clinical trial would be early terminated due to aforementioned reasons.

## Trial status

Study start date: August 1, 2021

Protocol version: 3

Anticipated completion date: December 2, 2022

## Data Availability

Final trial dataset will be made available to others. Data will be available from publication till 1 year. Any request for the data could be sent to ali.shaukat@duhs.edu.pk. There are no contractual agreements that can limit access of investigators to the final trial dataset.

## Abbreviations

ARDS: Acute respiratory distress syndrome
C-IVIG: Hyperimmune anti-COVID-19 intravenous immunoglobulin
CP: Convalescent plasma
CRF: Clinical research form
CRO: Clinical Research Organization
CTU: Clinical Trial Unit
DRAP: Drug Regulatory Authority Pakistan
SARS-CoV-2: Severe acute respiratory syndrome coronavirus 2
SE: Severe adverse events
SIDH: Sindh Infectious Disease Hospital
